# Novel Composite Nitride Nanoceramics from Reaction-Mixed Nanocrystalline Powders in the System Aluminum Nitride AlN/Gallium Nitride GaN/Titanium Nitride TiN (Al:Ga:Ti = 1:1:1)

**DOI:** 10.3390/ma15062200

**Published:** 2022-03-16

**Authors:** Mariusz Drygas, Katarzyna Lejda, Jerzy F. Janik, Svitlana Stelmakh, Bogdan Palosz

**Affiliations:** 1 Faculty of Energy and Fuels, AGH University of Science and Technology, al. Mickiewicza 30, 30-059 Krakow, Poland; madrygas@agh.edu.pl (M.D.); kkapusta@agh.edu.pl (K.L.); 2 Institute of High Pressure Physics, Polish Academy of Sciences, ul. Sokolowska 29/37, 01-142 Warszawa, Poland; svrit@unipress.waw.pl (S.S.); bogdan.palosz@unipress.waw.pl (B.P.)

**Keywords:** aluminium nitride AlN, gallium nitride GaN, aluminium gallium nitride AlGaN, titanium nitride TiN, nanopowders, sintering, composites, nanoceramics

## Abstract

A study is presented on the synthesis of reaction-mixed nitride nanopowders in the reference system of aluminium nitride AlN, gallium nitride GaN, and titanium nitride TiN (Al:Ga:Ti = 1:1:1) followed by their high-pressure and high-temperature sintering towards novel multi-nitride composite nanoceramics. The synthesis starts with a 4 h reflux in hexane of the mixture of the respective metal dimethylamides, which is followed by hexane evacuation, and reactions of the residue in liquid ammonia at −33 °C to afford a mixed metal amide/imide precursor. Plausible equilibration towards a bimetallic Al/Ga-dimethylamide compound upon mixing of the solutions of the individual metal-dimethylamide precursors containing dimeric {Al[N(CH_3_)_2_]_3_}_2_ and dimeric {Ga[N(CH_3_)_2_]_3_}_2_ is confirmed by ^1^H- and ^13^C{H}-NMR spectroscopy in C_6_D_6_ solution. The precursor is pyrolyzed under ammonia at 800 and 950 °C yielding, respectively, two different reaction-mixed composite nitride nanopowders. The latter are subjected to no-additive high-pressure and high-temperature sintering under conditions either conservative for the initial powder nanocrystallinity (650 °C, 7.7 GPa) or promoting crystal growth/recrystallization and, possibly, solid solution formation via reactions of AlN and GaN towards Al_0.5_Ga_0.5_N (1000 and 1100 °C, 7.7 GPa). The sintered composite pellets show moderately high mechanical hardness as determined by the Vicker’s method. The starting nanopowders and resulting nanoceramics are characterized by powder XRD, Raman spectroscopy, and SEM/EDX. It is demonstrated that, in addition to the multi-nitride composite nanoceramics of hexagonal AlN/hexagonal GaN/cubic TiN, under specific conditions the novel composite nanoceramics made of hexagonal Al_0.5_Ga_0.5_N and cubic TiN can be prepared.

## 1. Introduction

The artificially made metal and metalloid nitrides have been for sound reasons a focus of intensive research in recent years [1]. This is supported by the successful examples of such paramount applications of Group III nitrides as in efficient UV to blue range operating optoelectronic devices and white LEDs [2], by unmatched various ceramics/electronic applications for aluminium nitride AlN [3,4], boron nitride BN [5], silicon nitride Si_3_N_4_ [6,7], and titanium nitride TiN [8] or by the extensively studied applications of broad bandgap semiconductor gallium nitride GaN [1,2,9,10]. In this regard, composites made of the nitrides offer various attractive capabilities by utilizing the synergy of coexisting electronic and mechanical/thermal properties. This may include such properties as typical for semiconductor GaN with a broad bandgap of ca. 3.4 eV, characteristic high thermal conductivity and resistance of electrically insulating AlN, and an advantageous combination of tough mechanical and thermal resistance with extremely low electrical resistivity for TiN.

In particular, in the composite system of such three nitrides as AlN, GaN, and TiN, there is one more potential component to be considered, namely, AlGaN—a nitride “alloy”/solid solution of AlN and GaN known to exist in the entire compositional range. In our previous report on the preparation from organometallic precursors and sintering of nanopowders in the binary system of hexagonal AlN and hexagonal GaN, under selected conditions, the preparation of pure hexagonal Al_0.5_Ga_0.5_N nanoceramics was demonstrated for the first time [11]. The formation of the reaction-mixed AlN and GaN nanopowders in that system was observed to be accompanied by a partial reaction towards their solid solution, which was further enhanced upon high-pressure and high-temperature sintering (HP-HT sintering). The determined propensity for the AlGaN formation was linked to the specifics of the metals’ dimethylamide precursor chemistry in the bimetallic precursor solution. In particular, the formation was accelerated upon refluxing hexane solutions containing the mixture of the aluminum and gallium tris(dimethylamide) precursors in one of the preparation steps. The details of such chemistry are outlined in that report [11] and elsewhere [12].

In addition to the AlN-(AlGaN)-GaN system, we also reported on two more related binary systems, namely AlN-TiN and GaN-TiN, applying similar procedures as previously for composite nanopowder preparation, followed by HP-HT sintering towards the binary nitride nanoceramics. In the case of AlN-TiN, reaction-mixed powder composites made of hexagonal AlN and cubic TiN were sintered to composite nitride nanoceramics with high hardnesses comparable to those of the reference AlN and TiN ceramics [13]. The sintering was associated with phase segregation of hexagonal AlN and cubic TiN into relatively large distinct domains and, frequently, with formation of closed micro-sized pores linked mainly to the AlN component. No evidence was found for metastable solid solution formation of these two crystallographically different nitrides. Finally, for the binary system GaN-TiN, the chemically similar preparation of in situ made/mixed nitride nanopowders followed by HP-HT sintering afforded mechanically robust composite nanoceramics [14]. The study also confirmed quite extensive nitride phase separation in the micro-sized scale and no metastable solid solution formation on sintering of the stable hexagonal GaN and cubic TiN. In all the binary nitride systems, the impact of the average crystallite sizes of the reaction-mixed nanopowders as well as of applied sintering conditions (e.g., sintering temperature supporting or preventing crystal growth) was investigated and provided us with both general and specific patterns in such processing.

Herein, reported is a study aimed at the preparation of the ternary composite nanopowders of AlN, GaN, and TiN, including routes resulting in some solid solution AlGaN, and their high-pressure and high-temperature sintering towards composite nanoceramics under conditions retarding or enhancing crystal growth. Suitable modifications initially of the precursor chemistry and finally of the sintering stage provide a good deal of control over composition and mechanical properties of the nanoceramics. The application of high pressure is aimed at accelerating the sintering process and, together with applied high temperatures, both factors put definite constraints there on particle recrystallization if desired.

## 2. Experimental Section

### 2.1. Preparation of The Mixed Metal–Amide/Imide Precursor via Reflux in Hexane/Effective Equilibration of Ternary Solution in The Metal–Dimethylamide System {Al[N(CH_3_)_2_]_3_}_2_/{Ga[N(CH_3_)_2_]_3_}_2_/Ti[N(CH_3_)_2_]_4_, Atomic Ratio Al:Ga:Ti = 1:1:1

Samples of {Al[N(CH_3_)_2_]_3_}_2_ [15], 3.50 g (11.0 mmol of dimer), {Ga[N(CH_3_)_2_]_3_}_2_ [16], 4.45 g (11.0 mmol of dimer), and Ti[N(CH_3_)_2_]_4_ [17], 4.93 g (22.0 mmol of monomer) were synthesized according to the published procedures, dissolved together in 60 mL of dry hexane, and refluxed under an argon flow for 4 hrs. This was equivalent to at least 50% bimetallic dimer {Al/Ga[N(CH_3_)_2_]_3_}_2_, Al:Ga = 1:1 formation in the relevant binary system [12]. Subsequently, hexane was evacuated and liquid NH_3_ (ca. 60 mL) was transferred onto the residue, and the mixture was stirred under reflux at ca. –33 °C for 2 h, followed by a 2 h NH_3_ boil-off at this temperature. The resulting dark brown-colored solid was evacuated at RT for 0.5 h to yield homogeneously mixed polymeric Al/Ga/Ti-amide-imide precursor, stored under argon, for the preparation of composite nitride nanopowders.

### 2.2. Nitridation Pyrolysis

Trimetallic Al/Ga/Ti-amide-imide precursor was used in reactive pyrolysis preparations of composite nanopowders. The experiments were performed under a flow of NH_3_, 0.2 L/min, 4 hrs at two selected temperatures, 800 and 950 °C, for batches of the precursor loaded in an alumina boat. The products from both temperatures were dark brown-colored free flowing powders. 

### 2.3. No-Additive High-Pressure and High-Temperature Sintering

**Figure 1:**
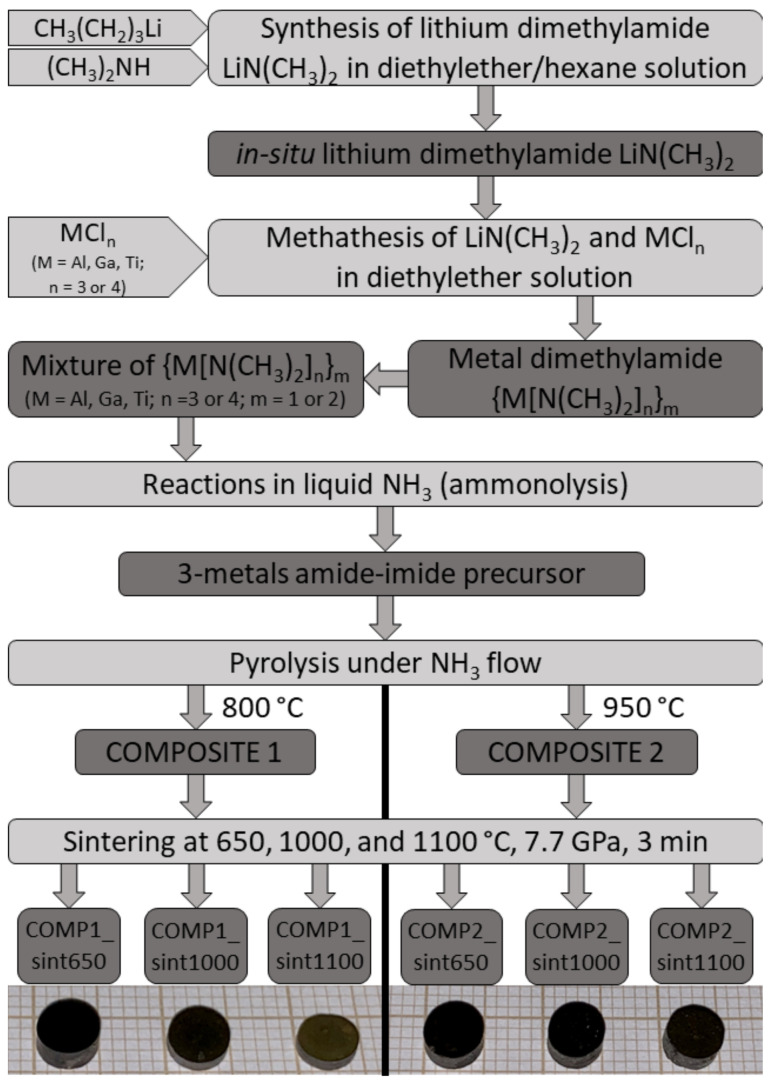
From chemical precursors to sintered nanoceramics—major steps of anaerobic synthesis and high-pressure and high-temperature sintering in the multi-nitride composite system AlN-GaN-TiN. Snapshots of sintered pellets are shown in the bottom part.

The powders prepared as described above were stored/handled under argon in a glovebox and, at some point, sealed in glass ampoules under vacuum. Upon ampoule opening, the powders were removed and briefly handled in air for loading prior to the high-pressure and high-temperature sintering using the methodology described elsewhere [18,19]. Specifically, the powders were sintered for 3 minutes in a high-pressure torroid cell at 650, 1000, and 1100 °C under a pressure of 7.7 GPa, yielding altogether six dark brown to black ceramic pellets, D = 5 mm, thickness ca. 3–4 mm. For Vicker’s hardness determinations under 300 g-force load, one of the pellet’s sides was polished. For other measurements, the pellets were coarsely ground in an agate mortar. The essential steps of powder preparation and sintering are shown in Figure 1. 

### 2.4. Nitride Sample Labeling

The composite nitride powders prepared from the mixed metal-amide-imide precursor upon pyrolysis at 800 and 950 °C are labelled, respectively, Composite 1 and Composite 2. The sintered ceramics have names of the related composite powder with suitable extensions for sintering temperature, e.g., Comp1_sint650 or Comp2_sint1000, etc.

### 2.5. Characterization

^1^H- and ^13^C{H}-NMR spectra were obtained using a Bruker Avance III 600 MHz spectrometer (Bruker, Billerica, MA, USA) at 300 K equipped with a nitrogen cryo-probe head. Spectra were recorded in C_6_D_6_ solutions that were contained in sealed 5 mm glass tubes, and the ^1^H and ^13^C chemical shifts were referenced to the residual solvent signals with TMS set to zero ppm. The spectra for the mixed metal-dimethylamides were acquired as soon as possible for (i) freshly prepared samples (spectra determined approx. 2 hours past metals dimethylamide 10-minute mixing in hexane solution at RT) and (ii) after the 3 h hexane reflux equilibration (spectra determined approx. 15 hours past metals dimethylamide mixing in hexane solution including the reflux and subsequent workout). Powder XRD determinations were carried out for all nitride nanopowders and ceramics on Empyrean PANalytical (Malvern, UK), Cu K_α_ source, 2Θ = 10–110°. Average crystallite sizes were evaluated from Scherrer’s equation applying the Rietveld refinement method. Due to extremely small crystallite sizes, the diffractions were often broadened and overlapped, resulting in crystallite cell parameter accuracies reported down to 0.01 Å. Raman spectroscopy was conducted with a WITec Alpha 300M+ spectrometer (WITec, Ulm, Germany) equipped with Zeiss optics (50×). Measurements were carried out using a 488 nm diode laser. Four accumulations of 30 s scans were gathered at each point. Baseline subtraction was performed using WITec’s software (ProjectFive Plus, WITec, Ulm, Germany). SEM/EDX data were acquired with a Hitachi Model S-4700 scanning electron microscope (Hitachi, Tokyo, Japan). The Vicker’s hardness (H_V_) tests were carried out on microhardness tester FutureTech FM-700 (Future-Tech Corp., Fujisaki, Japan) with a 300-gram force load, 10 s, and hardness was expressed in GPa.

## 3. Results and Discussion

### 3.1. From Molecular Precursors to Preparation of Nanopowders

The occurrence and progress of the three metal–dimethylamide interactions in hexane solution were investigated with ^1^H- and ^13^C{H}-NMR spectroscopy. At this stage, monomeric Ti-dimethylamide is not expected to interact with the other two dimeric metal-dimethylamides. On the other hand, the dimers of the Al- and Ga-dimethylamides are anticipated to react towards a mixed metal four-membered ring compound Al/Ga-dimethylamide according to Reaction (1) [12].

{Al[N(CH_3_)_2_]_3_}_2_ + {Ga[N(CH_3_)_2_]_3_}_2_ → 2{[CH_3_)_2_N]_2_Al[*µ*-N(CH_3_)_2_]_2_Ga[N(CH_3_)_2_]_2_} (1)

**Figure 2:**
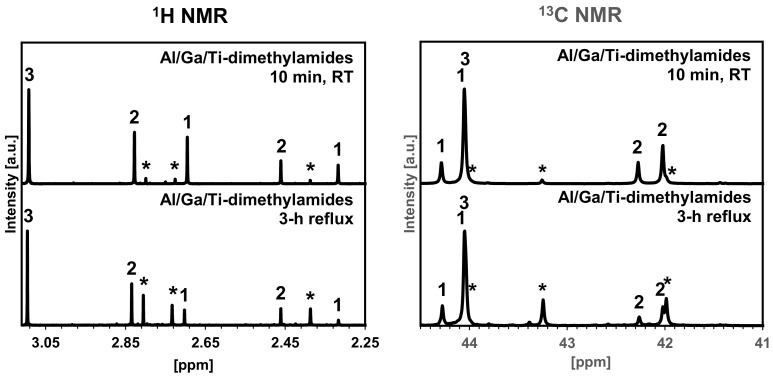
^1^H (left) and ^13^C (right) nuclear magnetic resonance (NMR) spectra in C_6_D_6_ of trimetallic solutions of Al/Ga/Ti-dimethylamides for short/inefficient (10 min, RT) and long/efficient (3 h reflux) equilibration in hexane. 1—two peaks for {Al[N(CH_3_)_2_]_3_}_2_; 2—two peaks for {Ga[N(CH_3_)_2_]_3_}_2_; 3—single peaks for Ti[N(CH_3_)_2_]_4_; asterisks—three peaks assigned to the bimetallic mixed metal four-membered-ring compound {[(CH_3_)_2_N]_2_Al[*µ-*N(CH_3_)_2_]_2_Ga[N(CH_3_)_2_]_2_} formed on equilibration.

In Figure 2, the results for the short 10-minute compound mixings in hexane solution at RT are compared with their 3-hour equilibration under reflux in hexane. In this regard, the two starting dimers of {Al[N(CH_3_)_2_]_3_}_2_ and {Ga[N(CH_3_)_2_]_3_}_2_ show each, both in the proton and carbon NMR spectra, two peaks with an intensity ratio 2:1 for the inequivalent terminal *exo*-N(CH_3_)_2_ (24H, 8C) and bridging *µ*-N(CH_3_)_2_ (12H, 4C) groups. Upon the dimers’ reaction, a mixed bimetallic four-membered ring compound {[(CH_3_)_2_N]_2_Al[*µ-*N(CH_3_)_2_]_2_Ga[N(CH_3_)_2_]_2_} is to be formed, showing three NMR peaks with intensities of 1:1:1 for the terminal *exo*-Al[N(CH_3_)_2_]_2_ (12H, 4C), bridging Al[*µ*-N(CH_3_)_2_]_2_Ga (12H, 4C), and *exo*-Ga[N(CH_3_)_2_]_2_ (12H, 4C) groups. At the same time, the third component in the solution, Ti[N(CH_3_)_2_]_4_, is monomeric and characteristic of the singlets in both the proton and carbon NMR spectra (24H, 8C). The NMR spectra confirm such a course of reaction and provide evaluation of the reaction progress. Specifically, the short 10-minute mixing time already results in some bimetallic Al/Ga-dimethylamide and integration of the relevant proton peak intensities for the two reacting compounds provided with the relative composition of 92% of the combined unreacted Al- and Ga-dimethylamide dimers and 8% of the mixed bimetallic product. The 3-hour reflux in hexane confirms the progression of the reaction towards the composition of approx. 51% of the unreacted dimers and 49% of the bimetallic product. The peaks assigned for the Ti-dimethylamide component do not undergo any changes, which is consistent with its chemically neutral behavior at this stage in the system. These observations agree well with the original work on the reactions in the binary Al- and Ga-dimethylamide system [12]. In conclusion, the 3-hour equilibration of the metal-dimethylamide substrates in refluxing hexane produces significant quantities of the bimetallic Al/Ga molecular component with {-N-Al-N-Ga-} linkages. The changes described above support a subsequent formation of the intimately mixed Al/Ga-amide-imide species on further ammonolysis reactions in liquid ammonia of such treated three metal-dimethylamides (Figure 1). Including the presence of Ti-dimethylamide, the ammonolysis is thought to result, eventually, in the domains of the Al-amide-imide [12,20], Ga-imide [21], Al/Ga-amide-imide [12], and Ti-imide [22] mixed on the molecular/nanosized level. These ammonolysis/transamination reactions are previously found to be facile for the individual and binary metal-dimethylamide systems and herein are anticipated to be equally efficient for the ternary metal-dimethylamide system to result in the three metal-amide-imide mixed precursor. Such a precursor is then converted to the reaction-mixed composite nitrides simply by pyrolysis under ammonia at two selected temperatures, i.e., 800 and 950 °C.

**Figure 3:**
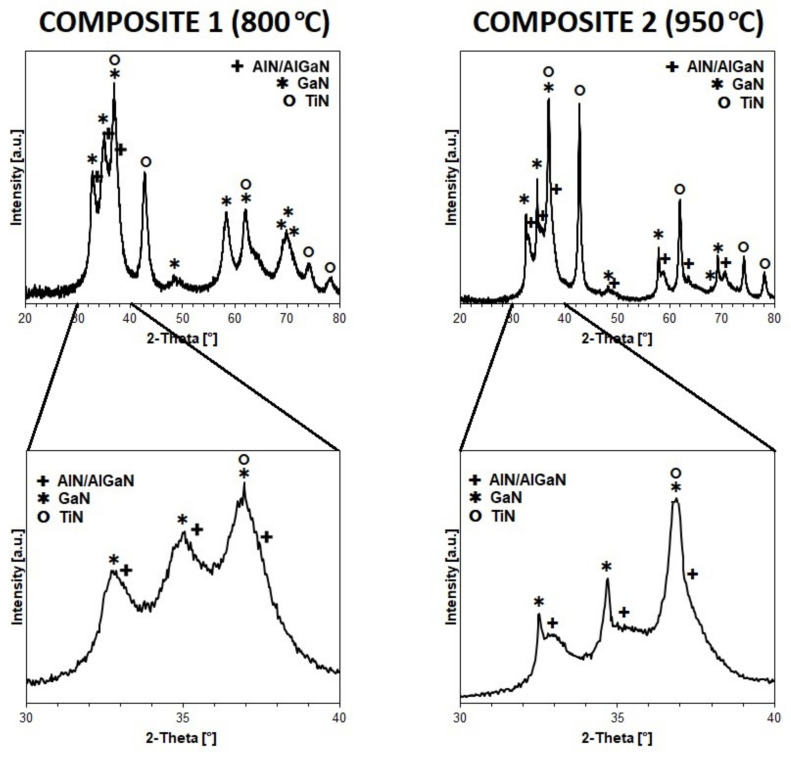
XRD patterns for 3-metal nitride Composite 1 (800 °C) and Composite 2 (950 °C). Bottom row shows expansion of the diagnostic 2-Theta 30–40° range. Assigned peaks are marked for hexagonal AlN/AlGaN (+), hexagonal GaN (*), and cubic TiN (°).

The XRD patterns for powder products from the nitriding pyrolysis at 800 °C (Composite 1) and 950 °C (Composite 2) are presented in Figure 3. The pattern for Composite 1 shows rather broad and superimposed peaks due to nanocrystallinity, especially of the hexagonal AlN/AlGaN and GaN components that have diffractions located in close vicinity. Some rather crude semiquantitative estimates for the pattern could be given by peak deconvolution but, particularly, the broadest peaks for AlN and, possibly, AlGaN are “buried” at other peaks bases, thus preventing any accurate peak width calculations. The presence of three nitride phases is elucidated, namely hexagonal GaN (a = 3.18 Å, c = 5.17 Å, D_av_ = 4 nm), tentative hexagonal “AlN/AlGaN” (a = 3.14 Å, c = 5.04 Å, D_av_ < 4 nm), and cubic TiN (a = 4.24 Å, D_av_ = 9 nm). The formation of some nanocrystalline AlGaN solid solution could only be speculated since its broad peaks are superimposed by equally broad peaks for AlN and they are tentatively deconvoluted together as “AlN/AlGaN”. Nonetheless, the slightly increased cell parameters for the hexagonal “AlN/AlGaN” phase vs. typical values for AlN made at this temperature, i.e., compare a = 3.14 Å, c = 5.04 Å in this study with a = 3.10 Å, c = 5.01 Å reported for the 800 °C-pyrolyzed pure AlN nanopowders [13], support some AlGaN presence. This can also be referenced to cell parameters reported for the monocrystalline form of hexagonal AlGaN, i.e., a = 3.1422(4) Å and c = 5.0842(5) Å [23]. Interestingly, when comparing the pattern with the relevant ones obtained for the similar composites prepared at 800 °C in the binary system made of Al-dimethylamide and Ga-dimethylamide [11] or Ga-dimethylamide and Ti-dimethylamide [14], the current pattern is consistent with distinctly worse crystallinity of the mixed nitrides. This can likely be due to a thinning effect of the additional third component TiN now present in the system. 

The pattern for Composite 2 supports its relatively better crystallinity that is manifested by higher average crystallite sizes of the nitride phases than found for Composite 1 as expected due to the higher nitridation temperature in the former case. The three nitride components are now quite clearly identified due to increased peak sharpness, especially for the TiN and GaN components, and provide the following parameters for hexagonal GaN (a = 3.19 Å, c = 5.19 Å, D_av_ = 21 nm), hexagonal “AlN/AlGaN” (a = 3.14 Å, c = 5.04 Å, D_av_ = 4 nm), and TiN (a = 4.24 Å, D_av_ = 19 nm). Additionally, again as in the case of Composite 1, a plausible formation of some AlGaN solid solution with pronounced nanocrystallinity cannot be excluded. It is to be recalled that in the binary system AlN-GaN, the XRD results for the 800 and 950 °C-prepared mixed nitrides also indicated some AlGaN formation [11]. In concluding the powder preparation section, two reaction-mixed 3-metal nitride composites, which differ in particle size characteristics of the component nitrides and may contain some AlGaN solid solution, are synthesized for sintering experiments.

### 3.2. From Reaction-Mixed Nitride Nanopowders to Sintered Composite Nanoceramics

The sintering experiments for both composite nanopowders were carried out under the pressure of 7.7 GPa for 3 minutes and at three temperature levels of 650, 1000, and 1100 °C, affording dark brown to black compact pellets of nanoceramics (Figure 1). The lowest temperature of 650 °C, being lower than the two nitridation temperatures used in composite preparation, was selected to secure sintering without crystal growth/recrystallization. The higher sintering temperatures of 1000 and 1100 °C exceeded the highest nitridation temperature of 950 °C and they were expected to promote crystal growth phenomena each at a different rate. These two sintering temperatures were also in the range of GaN nanopowder thermal stability [24], but the short sintering time was found to limit GaN decomposition quite effectively [14]. It is to recall that the application of high pressure was, first, to accelerate sintering while counterbalancing to some extent the temperature induced crystal growth and, second, it was expected to promote further AlGaN formation via reactions of available AlN and GaN. 

**Figure 4:**
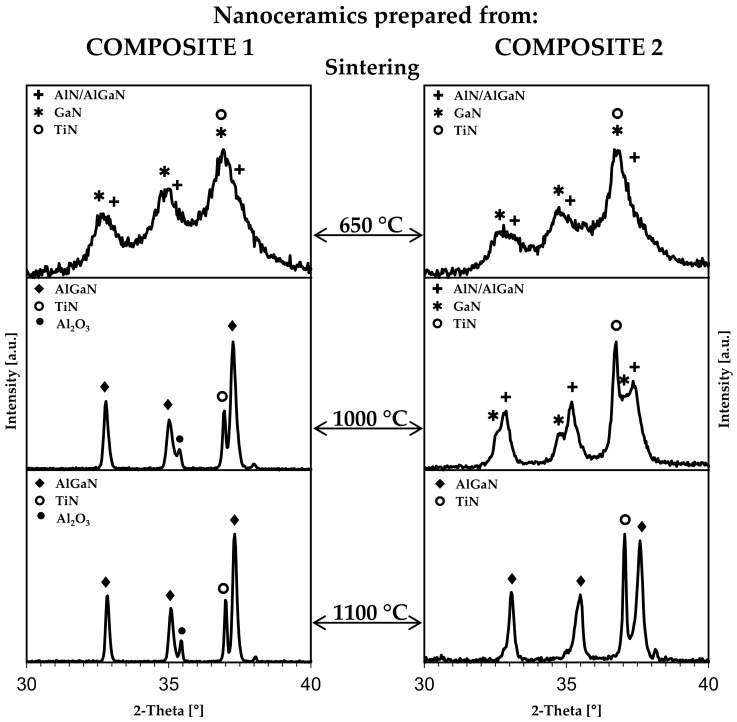
XRD patterns in the 2-Theta range of 30–40° for pellets sintered at temperatures 650, 1000, and 1100 °C from Composite 1 (left column) and Composite 2 (right column). For pellets of Composite 1 sintered at 1000 and 1100 °C, small quantities of the order of several percent of adventitious Al_2_O_3_ (•) are detected on the surface.

The XRD patterns in the diagnostic 2-Theta range of 30–40° for the pellets are shown in Figure 4. The calculated cell parameters and average crystallite sizes are included in Table 1. It appears by comparing Figure 3 and Figure 4 that sintering at 650 °C does not cause any significant phase changes in the system. First, this temperature is lower than the nitride composite preparation temperatures of 800 and 950 °C, and the process is carried out without recrystallization. Second, the prevailing in such a case action of the high pressure is reflected by the decreased average crystallite diameters as clearly seen for the well-resolved cubic TiN component, i.e., for Composite 1, the D_av_ decreases from 9 in nanopowder to 8 nm in nanoceramics and for Composite 2 from 19 to 15 nm, respectively. The remaining nitrides in the mixture that include hexagonal GaN and tentative “AlN/AlGaN” appear to remain mostly unchanged. Due to high uncertainty of peak deconvolution results, they are not calculated, but the earlier discussion concerned with the substrate composites qualitatively holds also true in this case. When applying the higher sintering temperatures of 1000 and 1100 °C, the pronounced changes take place—the AlN and GaN components efficiently react to form now a bimetallic hexagonal phase of AlGaN, or more precisely Al_0.5_N_0.5_N, in the composite system with cubic TiN. 

It is also worth pointing out that the combined effects of each of the two high-temperature levels (1000 and 1100 °C) and very high pressure in sintering result in the smaller average crystallite sizes of all nitride phases for Composite 2-derived nanoceramics compared to Composite 1 nanoceramics (Table 1). This is despite the fact that the starting composite nanopowders are characterized by a reverse order of the nitride phase sizes, i.e., the sizes are considerably higher for nanopowders of Composite 2 compared to Composite 1, as discussed earlier. This can be explained/understood in terms of the temperature difference between the nanopowder preparation and sintering stages and its impact on crystal growth during sintering. For Composite 1 prepared at 800 °C, the difference is 200 or 300 °C, whereas for Composite 2 made at 950 °C, it is 50 or 150 °C considering the sintering temperatures of 1000 or 1100 °C, respectively. The relatively higher temperature difference levels for Composite 1 constitute a more efficient driving force for crystal growth/recrystallization of the nitride phases than occurring for Composite 2 under the applied pressure and time conditions. Additionally, for a smaller temperature spread between the powder preparation and sintering temperatures, the crystallite “crushing” effect of the applied high pressure comes to play a more pronounced role [11,13,14], adding to a net size effect.

The temperature levels are sufficiently high to enable the formation of the AlGaN solid solution which is, additionally, coupled with the nitrides’ crystal growth into the average crystallite sizes up to several tens of nanometer. The crystallographic cell parameters for the hexagonal AlGaN and cubic TiN are found to be identical for nanoceramics of Comp1_sint1000 and Comp1_sint1100, whereas the average crystallite sizes are higher for the higher sintering temperature, and this is consistent with temperature-induced crystal growth. Very similar trends apply to Composite 2-derived nanoceramics of Comp2_sint1000 and Comp2_sint1100, although the cell parameters for hexagonal AlGaN differ slightly from their counterparts made from Composite 1. 

It is interesting to compare the cell parameters for hexagonal Al_0.5_Ga_0.5_N prepared in this study with the earlier reported data for such a solid solution made in the binary system AlN-GaN, i.e., a = 3.175 Å, c = 5.151 Å [11]. It appears that the two sets are very close confirming that the nanoceramics, which are reactively formed under extreme high-pressure and high-temperature conditions, may have favoured a specific metastable structure of hexagonal Al_0.5_Ga_0.5_N. In concluding this section, a novel composite nanoceramic made of hexagonal Al_0.5_Ga_0.5_N and cubic TiN can be prepared via reactive sintering of the suitable composite nitride nanopowders.

**Figure 5:**
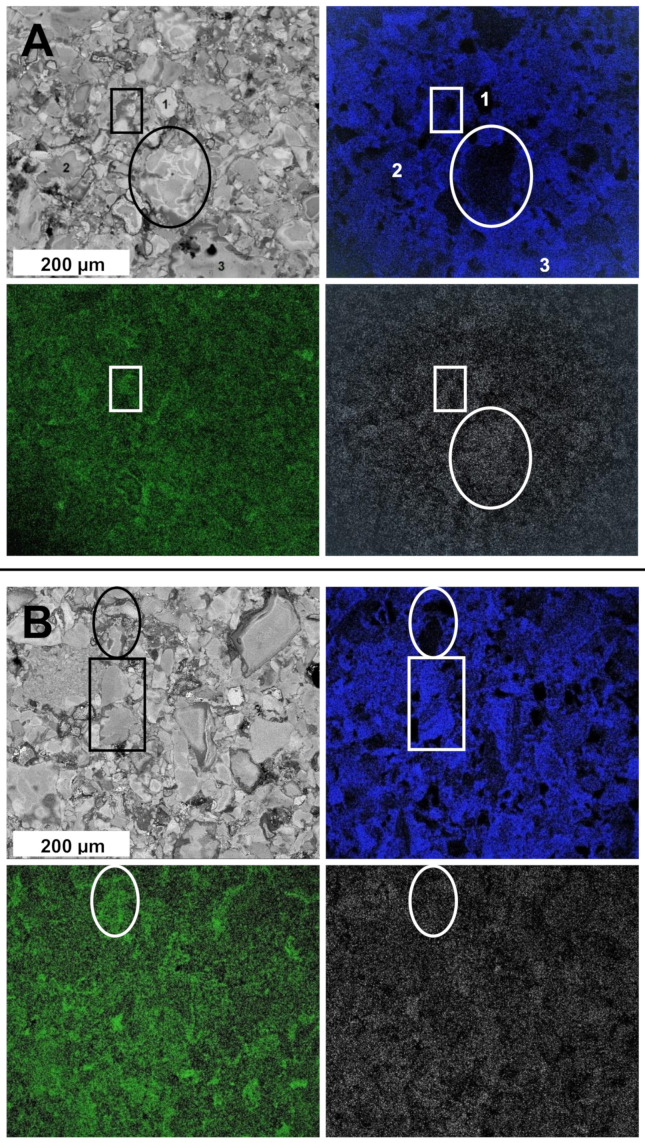
SEM/EDX images of pellets for (**A**)—Comp2_sint650 and (**B**)—Comp2_sint1000 including elemental maps for Al (green), Ga (grey), and Ti (blue). For (**A**), spots labelled 1, 2, and 3 were analysed by EDX.

The SEM/EDX examination of the polished surfaces of pellets, shown in Figure 5, is exemplified by the case of Comp2_sint650 and Comp2_sint1000, with the former pellet being sintered without recrystallization and the latter with recrystallization. For Comp2_sint650, there are clearly present prevailing microsized domains of, especially, GaN and TiN, which support compound phase segregation on sintering. The area enclosed in oval corresponds to such a domain of GaN as seen from comparison of the maps for Ti (blue) and Ga (grey), and the SEM image.

The rectangular area is associated with predominance of Ga and Al species over Ti. The map for Al (green) indicates much smaller AlN domains forming a matrix for the more extensively segregated GaN and TiN. The EDX point elemental analysis supports the black area (labelled with spot 1) in the Ti’s map to be enriched mostly in Ga and, possibly, Al, whereas the bluish areas of the spots 2 and 3 are to be enriched in Ti. For the Comp2_sint1000 pellet (sintering with recrystallization), the maps indicate progression of phase segregation; now with the TiN domains relatively better shaped up, the domains in the blue map for Ti and Ga/Al appear to be more shape-defined as supported both by the rectangular (Ti) and oval (Ga/Al) areas. In this case, the features of the Al and Ga maps are very similar to each other which is consistent with the AlGaN solid solution present in these ceramics. Additionally, a careful inspection of the pellet’s surface in the SEM image supports existence of noticeable microsized porosity—a feature described earlier for the relevant, while simpler, binary nitride nanoceramics [11,13,14]. This could be a side effect of diffusion-assisted crystal growth phenomena operating during the specific HP-HT sintering process.

**Figure 6:**
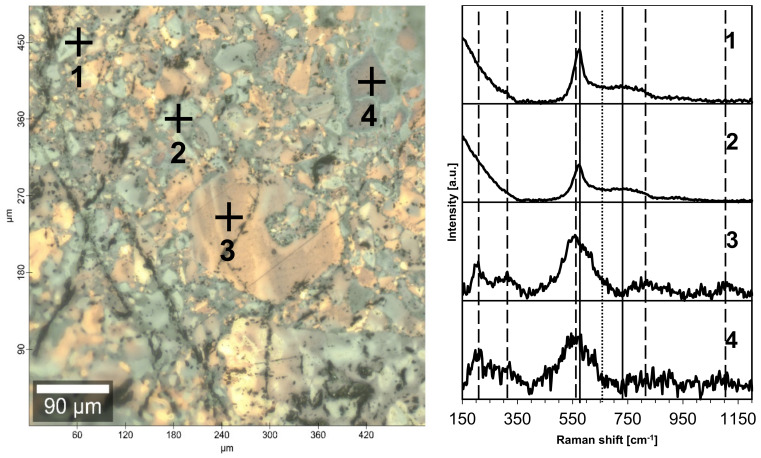
Micro-Raman spectra for nanoceramics Comp1_sint650 collected at points 1 through 4 (see microscopic image of the pellet’s polished surface on the left). Vertical lines are guides for eye only, and they are placed in positions of various modes of AlN (dotted), GaN (solid), and TiN (dashed).

Raman spectroscopy equipped with confocal microscopy can also be used to investigate composite nanoceramics from the angle of phase domains. It is instructive to recall that the first-order Raman spectra are forbidden for perfect O_h_ symmetry in cubic TiN. However, due to the high propensity of the nitride for nonstoichiometry with nitrogen deficiency and the then-active modes operating, Raman spectroscopy is frequently used for such titanium nitride products as single crystals [25], thin layers [26], and powders [27]. The case of Comp1_sint650 shown in Figure 6 confirms the notion that even sintering without recrystallization leads also to segregated microsized phase domains. First, the various domains are differentiated by color and the light beige to golden regions are confirmed to be TiN, the darker bluish regions correspond to GaN, and the dark grey to black areas are enriched in AlN. Second, for regions 1 and 2, the spectra have two major bands, i.e., 570–580 cm^−1^ (sharp) and 720–730 cm^−1^ (broad), that are typical for the GaN bands of E_2_ (high) and A_1_ (LO), respectively [28,29]. Regions 3 and 4 show the bands typical for TiN including square-like acoustic phonons with TA and LA modes at ca. 220 cm^−1^ to 320 cm^−1^, strong TO mode at 560 cm^−1^, and two wide maxima of overtones at ca. 820 (LA + TO) and 1120 cm^−1^ (2 TO), the latter typical for nearly stoichiometric TiN [30]. It is worth noticing that the presence of the second-order overtones is consistent with a relatively small N deficiency in the titanium nitride domains [31]. Additionally, third, the domains of extremely nanosized AlN are quite well distributed throughout the entire volume of the pellet so the nitride’s characteristic E_2_ (high) band at ca. 655 cm^−1^ [32] remains largely undetected. 

It is likely that the band contributes to the broad neck between the two GaN bands for regions 1 and 2. Finally, a very similar picture of domain compositions is found in the Raman spectrum for Comp2-sint650 (not shown). In concluding this section, the Raman spectroscopy confirms the segregation and extensive aggregation of the nitrides’ phases during the HP-HT sintering even in the absence of temperature-induced phase recrystallization.

The helium density d_He_ and Vicker’s hardness H_V_ data are included in Table 2. Regarding helium density, the measured values of d_He_ can be compared to the theoretical density 4.91 g/cm^3^ of a 1:1:1 composite of AlN, GaN, and TiN (molar basis) assuming the pure nitrides theoretical densities of 3.26, 6.15, and 5.24 g/cm^3^, respectively. The values of % theor. in the table reflect the comparison in terms of percentages. It is apparent that the composite densities are relatively high, i.e., above 95%. In two cases, they exceed slightly 100%, i.e., 102 and 103%, and this is likely the effect of some pellet surface oxidation confirmed by the XRD evidence of small quantities of Al_2_O_3_ with a density of 3.95 g/cm^3^ (Figure 4). 

It is instructive to compare this with similarly measured densities in the binary composite systems of AlN-GaN—84–95% [11], AlN-TiN—70–82% [13] and GaN-TiN—74–81% [14]. The data have to be treated as approximate since in a few cases one does not deal with simple nitrides but, instead, with AlGaN solid solutions. Nonetheless, the distinctly high helium densities of the pellets in this study point out to the efficient compaction in HP-HT sintering and relatively low remnant closed porosity. The reactive formation of AlGaN in such a component system, also observed in the binary system AlN-GaN [11], appears to favour achieving very high densities of the resultant composite nanoceramics.

The Vicker’s hardness values for the ternary composite nanoceramics are moderately high, ranging from 10.6 to 13.5 GPa, and can be referenced to the available data both for the individual/pure and binary sintered nitride systems studied by us and others. For pure hexagonal AlN nanoceramics, we reported H_V_ spanning 12.4–17.4 GPa [13], while others quoted values from 17.7 [33] up to a theoretical limit of 20 GPa [34]. For cubic TiN nanoceramics, a range of 13.5–19.7 GPa was reported [13] and a few micrometer-thick layers of TiN were shown to exhibit hardness H_V_ from 9.2 to 15.0 GPa [35]. In this regard, for the binary system AlN-TiN, the H_V_s were found in the range 6.7–19.9 GPa [13]. The pure GaN nanoceramics provided H_V_s from10.0 to 17.4 GPa, whereas in the binary system GaN-TiN, H_V_s from 7.5 to 13.2 GPa were reported [14]. Interestingly, the Vicker’s hardness values for pure GaN nanoceramics in our studies were generally higher than the reference literature values for GaN of 11 GPa [33]. A significant trend in the nitrides’ hardness was observed by us in the binary system AlN-GaN with the formation of the novel nanoceramics Al_0.5_Ga_0.5_N—the nanoceramics made of pure solid solution were shown to have a rather low H_V_ of 3.8 GPa, and another made mostly of Al_0.5_Ga_0.5_N was shown to have a bit higher, but still low, H_V_ of 7.7 GPa [11]. The decreased hardness of such ceramics was proposed to result from the evolution of an extensive net of microsized pores that accompanied, by way of extensive mass diffusion, the reactive formation of the solid solution during sintering. It is worth pointing out that in the current study, the lowest H_V_ of 10.6 GPa, while moderately high, is recorded for the novel nanoceramics made of hexagonal Al_0.5_Ga_0.5_N and cubic TiN, which is consistent with the somewhat negative impact of the solid solution in situ formation on hardness.

## 4. Conclusions

The idea of a reaction-mixed preparation of composite nitride nanopowders from molecular precursors is confirmed for a 3-metal nitride composites in the reference system AlN-GaN-TiN. In particular, the propensity of the starting Al- and Ga-dimethylamides to react towards a bimetallic Al/Ga-dimethylamide compound with intermixed metal sites {-N-Al-N-Ga-} was studied and utilized to lead, eventually, to some binary mixed metal AlGaN components (solid solution of AlN and GaN) in the composite nitride nanopowders. No-additive high-pressure and high-temperature sintering of such nanopowders, with or without recrystallization, has been shown under selected conditions to result in mechanically robust nanoceramics with compositions ranging from AlN-AlGaN-GaN-TiN to Al_0.5_Ga_0.5_N-TiN, Al:Ga:Ti = 1:1:1 (molar ratio). An interesting phenomenon of the nitride phase segregation/separation during sintering is observed even under conditions of sintering without recrystallization. The composite nanoceramics made of hexagonal Al_0.5_Ga_0.5_N and cubic TiN is demonstrated for the first time, confirming the utility of the molecular precursor approach. The relatively high helium densities (>95% of theoretical) and moderately high Vicker’s hardness (10.6–13.5 GPa) of the nanoceramics constitute advantageous features of the discussed nanopowder preparation and sintering scheme.

## Figures and Tables

**Table 1:** Crystallographic cell parameters *a* and *c* and average crystallite sizes D_av_ estimated from XRD patterns for composite nanoceramics in the system AlN-GaN-TiN sintered at 650, 1000, and 1100 °C.

Composite Nanoceramics from:	Sintering Temperature				
	650 °C		1000 °C		1100 °C
COMPOSITE 1:	Comp1_sint650		Comp1_sint1000		Comp1_sint1100
(i) hexagonal phase:	GaN	“AlN/AlGaN”	Al_0.5_Ga_0.5_N		Al_0.5_Ga_0.5_N
a (Å)	3.20	3.17	3.18		3.18
c (Å)	5.19	5.11	5.16		5.16
D_av_ (nm)	6	–	58		77
(ii) cubic phase:	c-TiN		c-TiN		c-TiN
a (Å)	4.24		4.24		4.24
D_av_ (nm)	8		93		182
COMPOSITE 2:	Comp2_sint650		Comp2_sint1000		Comp2_sint1100
(i) hexagonal phase:	GaN	“AlN/AlGaN”	GaN	”AlN/AlGaN”	Al_0.5_Ga_0.5_N
a (Å)	3.18	3.13	3.18	3.15	3.17
c (Å)	5.17	5.06	5.15	5.10	5.13
D_av_ (nm)	7	–	51	23	53
(ii) cubic phase:	c-TiN		c-TiN		c-TiN
a (Å)	4.24		4.24		4.25
D_av_ (nm)	15		43		86

**Table 2:** Helium density d_He_ and Vicker’s hardness H_V_ (300 g-force load) data including standard deviations SD for composite nanoceramics in the reference system AlN-GaN-TiN. Percentages % theor. were calculated with respect to theoretical density 4.91 g/cm^3^ of mixed nitride composite AlN:GaN:TiN = 1:1:1 (molar basis).

Composite Nanoceramics from:	Sintering Temperature		
	650 °C	1000 °C	1100 °C
COMPOSITE 1 (800 °C)			
d_He_ (SD) (g/cm^3^):	4.66 (0.003)	5.02 (0.002)	5.04 (0.003)
% theor.:	95	102	103
H_V_ (SD) (GPa):	13.5 (1.3)	12.1 (0.7)	12.2 (2.4)
COMPOSITE 2 (950 °C)			
d_He_ (SD) (g/cm^3^):	4.71 (0.003)	4.68 (0.006)	4.84 (0.004)
% theor.:	96	95	98
H_V_ (SD) (GPa):	10.8 (1.7)	12.1 (1.9)	10.6 (1.1)

## Data Availability

The data presented in this study are available on request from the corresponding author.
